# Practical Oral Hygiene, Prophylaxis and Pyorrhea Alveolaris

**Published:** 1915-09

**Authors:** 


PRACTICAL ORAL HYGIENE, PROPHYLAXIS AND PYORRHEA ALVEOLARIS. By Robin Adair, B.S., M.D., D.D.S. This new and greatly enlarged edition of this work is just published by the Oral Hygiene Publishing Co., of Atlanta, Ga The former book has been materially enlarged so that the present edition numbers nearly 500 pages. The press work of the present edition is a great improvement on the former edition, which makes the volume more comfortable to read. The illustrations are abundant and well selected.

The work is subdivided for systematic consideration into three parts under these headings: I, "Practical Oral Hygiene," being a consideration of the general public work of oral hygiene, such as school clinics, lectures, etc. Part II, "Practical Oral Prophylaxis," being devoted to a consideration of the professional practice of oral hygiene. Part III, "Pyorrhea Alveolaris and Its Treatment." This is an effort to record present knowledge of the etiology and various treatments advocated for its cure, both of a surgical and medicinal character.

The book is a most interesting collection of the general discussion and effort along this line of dental activity for the past few years. It does not attempt to harmonize views and to dogmatize as to practice, and we are tempted to compliment the author on his skillful efforts to present the great variety of views as to cause and treatment for both the preventive and rescue treatments. He certainly has kept in touch with the work and has made a remarkably successful compilation of what has been done. At the same time the author has perhaps too modestly reported his own methods and conclusions. It is a book that every dentist will be pleased to read, and every one can read it with profit. On this subject we are not yet ready for a scientific treatise.

